# Bis[μ-3-(1*H*-pyrazol-1-yl)benzonitrile-κ^2^ *N*:*N*′]bis­[perchloratosilver(I)]

**DOI:** 10.1107/S1600536808030602

**Published:** 2008-09-27

**Authors:** Cao-Yuan Niu, Hai-Yan Zhang, Cao-Ling Feng, Xin-Sheng Wan, Chun-Hong Kou

**Affiliations:** a College of Sciences, Henan Agricultural University, Zhengzhou 450002, People’s Republic of China

## Abstract

In the title centrosymmetric complex, [Ag_2_(ClO_4_)_2_(C_10_H_7_N_3_)_2_], the unique Ag^I^ ion is coordinated by an N atom from a carbonitrile group, an N atom from a symmetry-related pyrazole group and an O atom of a perchlorate ligand to form a distorted T-shaped environment. Two 3-(1*H*-pyrazol-1-yl)benzonitrile ligands each bridge two Ag^I^ ions to form a dinuclear complex. In the crystal structure, there are weak Ag⋯O inter­actions within the range 2.70–3.01 Å linking dimeric units into layers approximately parallel to (100). The O atoms of the perchlorate ligand are disordered over two sites with occupancies of 0.570 (11) and 0.430 (11), respectively.

## Related literature

For background information, see: Antonioli *et al.* (2006 ▶); Bourlier *et al.* (2007 ▶); Niu *et al.* (2007 ▶); Sumby & Hardie (2005 ▶).
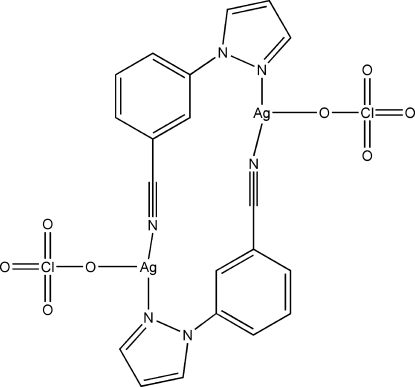


## Experimental

### 

#### Crystal data

[Ag_2_(ClO_4_)_2_(C_10_H_7_N_3_)_2_]
*M* *_r_* = 753.02
Monoclinic, 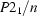

*a* = 7.8522 (13) Å
*b* = 10.6086 (17) Å
*c* = 15.322 (2) Å
β = 101.100 (2)°
*V* = 1252.5 (3) Å^3^
*Z* = 2
Mo *K*α radiation
μ = 1.83 mm^−1^
*T* = 173 (2) K
0.51 × 0.47 × 0.36 mm

#### Data collection

Siemens SMART CCD diffractometer
Absorption correction: multi-scan (*SADABS*; Sheldrick, 1996 ▶) *T*_min_ = 0.455, *T*_max_ = 0.558 (expected range = 0.421–0.517)
7721 measured reflections
2833 independent reflections
2180 reflections with *I* > 2σ(*I*)
*R*_int_ = 0.021

#### Refinement

*R*[*F* ^2^ > 2σ(*F* ^2^)] = 0.042
*wR*(*F* ^2^) = 0.114
*S* = 0.96
2833 reflections
209 parameters
74 restraints
H-atom parameters constrained
Δρ_max_ = 0.75 e Å^−3^
Δρ_min_ = −0.59 e Å^−3^

### 

Data collection: *SMART* (Siemens, 1996 ▶); cell refinement: *SAINT* (Siemens, 1994 ▶); data reduction: *SAINT*; program(s) used to solve structure: *SHELXS97* (Sheldrick, 2008 ▶); program(s) used to refine structure: *SHELXL97* (Sheldrick, 2008 ▶); molecular graphics: *SHELXTL* (Sheldrick, 2008 ▶) and *PLATON* (Spek, 2003 ▶); software used to prepare material for publication: *SHELXTL*.

## Supplementary Material

Crystal structure: contains datablocks I, global. DOI: 10.1107/S1600536808030602/lh2676sup1.cif

Structure factors: contains datablocks I. DOI: 10.1107/S1600536808030602/lh2676Isup2.hkl

Additional supplementary materials:  crystallographic information; 3D view; checkCIF report

## Figures and Tables

**Table:** 

Ag1—N3^i^	2.154 (6)
Ag1—N1	2.198 (4)
Ag1—O4	2.495 (6)
Ag1—O4′^ii^	2.609 (6)

**Table:** 

N3^i^—Ag1—N1	147.16 (17)
N3^i^—Ag1—O4	105.89 (19)
N1—Ag1—O4	89.96 (19)
N1—Ag1—O4′^ii^	98.4 (19)
N3^i^—Ag1—O4′^ii^	110.8 (19)

Symmetry codes: (i) 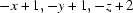; (ii) 
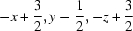
.

**Fig. 1.**
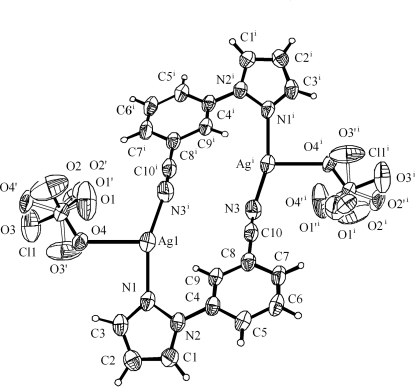
A view of the AgI coordination environment in the dimeric structure of (I), showing the atom-labelling scheme. Displacement ellipsoids are drawn at the 30% probability level and H atoms are shown as small spheres of arbitrary radii. [Symmetry codes: (i) -x + 1, -y + 1, -z + 2] The minor component of disorder is shown with open bonds.

**Fig. 2.**
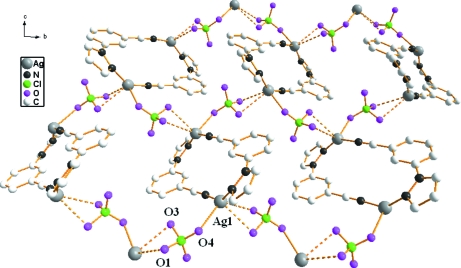
Part of the crystal structure of (I). Dashed lines show weak Ag···O interactions. The minor component of disorder is not shown.

**Fig. 3.**
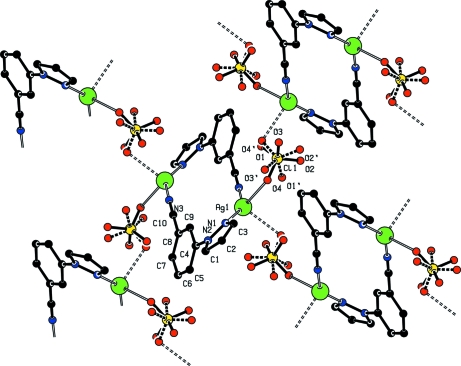
Part of the crystal structure showing the major and minor (dashed bonds) components of disorder.

## supplementary crystallographic information

### Comment

Silver coordination polymers have been widely studied not only for their utility in special functional materials, but also for their fascinating structures derived from variable coordination numbers from 2 to 6 of silver atoms and different conformations around silver metal centres (Sumby & Hardie, 2005; Niu *et al.*, 2007). Nitrogen heterocycle organic compounds with multiple pyridyl, or pyrazole, or carbonitrile nitrogen atoms are good bridging organic ligands in coordination interactions with silver atoms (Antonioli *et al.*, 2006; Bourlier *et al.*, 2007). Herein we communicate the crystal structure of one silver coordination dimer with one asymmetric organic bridging ligand, 3-(1*H*-pyrazol-1-yl)benzonitrile, with carbonitrile and pyrazole coordinating groups.

In the title compound, (I), the central silver ion is coordinated by two nitrogen atoms [N1, N3^i^; Symmetry codes: (i) -*x* + 1, -*y* + 1, -*z* + 2] of carbonitrile and pyrazole groups from two different 3-(1*H*-pyrazol-1-yl)benzonitrile molecule and one oxygen atom [O4] from one perchlorate anion, forming the primary distorted T-shape coordination environment around the silver atom (Fig .1). The O atoms of the perchlorate ligand are disodered over two sites with maximum and minimum occupancies of 0.57 and 0.43. While an O atom of the major component coordinates to the unique Ag^I^ ion, an O atom of the minor component coordinates to a symmetry related Ag^I^ ion. The overall effect of the disorder is that two different slightly distorted T-shaped coordination enviroments are formed with the two Ag—O disorder components being approximately orthogonal to each other (Fig. 3).

In (I), the 3-(1*H*-pyrazol-1-yl)benzonitrile molecule ligand acts as a µ_2_-bridging ligand. Two ligands each bridge two metal centres through one carbonitrile nitrogen atom and one pyrazole nitrogen atom to form a small centrosymmetric 2+2 Ag_2_*L*_2_ (*L* = ligand) ring as a constructing unit (Fig. 1). The Ag···Ag separation in one ring is about 6.852 (5) Å. There are weak Ag···O interactions between Ag1 and O1 and Ag1 and O3 with the separations of about 2.89 and 3.01 Å, respectively [Ag1···O1' = 2.70 Å]. Supramolecular two-dimensional layers are constructed through the strong Ag—O bonds and weak Ag···O interactions between perchlorate anions and silver atoms of dinuclear rings (Fig. 2). The layers lie approximately parallel to the *b**c* plane.

### Experimental

A solution of AgClO_4_.H_2_O (0.023 g, 0.1 mmol) in methanol (10 ml) was carefully layered on a methanol/chloroform solution (5 ml/10 ml) of 3-(1*H*-pyrazol-1-yl)benzonitrile (0.017 g, 0.1 mmol) in a straight glass tube. About one week later, colourless single crystals of (I) suitable for X-ray analysis were obtained (yield 39%).

### Refinement

Carbon-bound H atoms were placed in calculated positions and refined using a riding model, with C—H = 0.95 Å and *U*_iso_(H) = 1.2*U*_eq_(C). The oxygen atoms of the perchlorate anion are disordered over two positions. All Cl—O bond lengths were restrained to 1.44 (1) Å. The final difference Fourier map had a highest peak at 0.84 Å from atom O4 and a deepest hole at 0.73 Å from atom Ag1, but were otherwise featureless.

### Crystal data

**Table:**

[Ag_2_(ClO_4_)_2_(C_10_H_7_N_3_)_2_]	*F*(000) = 736
*M**_r_* = 753.02	*D*_x_ = 1.997 Mg m^−^^3^
Monoclinic, *P*2_1_/*n*	Mo *K*α radiation, λ = 0.71073 Å
Hall symbol: -P 2yn	Cell parameters from 3269 reflections
*a* = 7.8522 (13) Å	θ = 2.7–27.5°
*b* = 10.6086 (17) Å	µ = 1.84 mm^−^^1^
*c* = 15.322 (2) Å	*T* = 173 K
β = 101.100 (2)°	Prism, colourless
*V* = 1252.5 (3) Å^3^	0.51 × 0.47 × 0.36 mm
*Z* = 2	

### Data collection

**Table:**

Siemens SMART CCD diffractometer	2833 independent reflections
Radiation source: fine-focus sealed tube	2180 reflections with *I* > 2σ(*I*)
graphite	*R*_int_ = 0.021
φ and ω scans	θ_max_ = 27.5°, θ_min_ = 2.7°
Absorption correction: multi-scan (SADABS; Sheldrick, 1996)	*h* = −10→10
*T*_min_ = 0.455, *T*_max_ = 0.558	*k* = −13→9
7721 measured reflections	*l* = −17→19

### Refinement

**Table:**

Refinement on *F*^2^	Primary atom site location: structure-invariant direct methods
Least-squares matrix: full	Secondary atom site location: difference Fourier map
*R*[*F*^2^ > 2σ(*F*^2^)] = 0.042	Hydrogen site location: inferred from neighbouring sites
*wR*(*F*^2^) = 0.114	H-atom parameters constrained
*S* = 0.96	*w* = 1/[σ^2^(*F*_o_^2^) + (0.0532*P*)^2^ + 1.7339*P*] where *P* = (*F*_o_^2^ + 2*F*_c_^2^)/3
2833 reflections	(Δ/σ)_max_ < 0.001
209 parameters	Δρ_max_ = 0.75 e Å^−^^3^
74 restraints	Δρ_min_ = −0.59 e Å^−^^3^

### Special details

**Table:**

Geometry. All e.s.d.'s (except the e.s.d. in the dihedral angle between two l.s. planes) are estimated using the full covariance matrix. The cell e.s.d.'s are taken into account individually in the estimation of e.s.d.'s in distances, angles and torsion angles; correlations between e.s.d.'s in cell parameters are only used when they are defined by crystal symmetry. An approximate (isotropic) treatment of cell e.s.d.'s is used for estimating e.s.d.'s involving l.s. planes.
Refinement. Refinement of *F*^2^ against ALL reflections. The weighted *R*-factor *wR* and goodness of fit *S* are based on *F*^2^, conventional *R*-factors *R* are based on *F*, with *F* set to zero for negative *F*^2^. The threshold expression of *F*^2^ > σ(*F*^2^) is used only for calculating *R*-factors(gt) *etc*. and is not relevant to the choice of reflections for refinement. *R*-factors based on *F*^2^ are statistically about twice as large as those based on *F*, and *R*- factors based on ALL data will be even larger.

### Fractional atomic coordinates and isotropic or equivalent isotropic displacement parameters (Å^2^)

**Table:**

	*x*	*y*	*z*	*U*_iso_*/*U*_eq_	Occ. (<1)
Ag1	0.76740 (6)	0.39146 (4)	0.86430 (3)	0.07794 (19)	
N1	1.0182 (5)	0.3203 (4)	0.9364 (2)	0.0701 (10)	
N2	1.0408 (5)	0.2463 (4)	1.0104 (2)	0.0651 (9)	
N3	0.4622 (7)	0.5000 (5)	1.1304 (3)	0.0911 (14)	
Cl1	0.84901 (17)	0.60883 (10)	0.70970 (7)	0.0661 (3)	
O1	0.7551 (17)	0.6867 (11)	0.7615 (9)	0.135 (6)	0.570 (11)
O2	0.7442 (18)	0.5787 (12)	0.6285 (7)	0.202 (8)	0.570 (11)
O3	0.9945 (14)	0.6862 (10)	0.6941 (9)	0.176 (6)	0.570 (11)
O4	0.9186 (10)	0.5048 (6)	0.7585 (5)	0.086 (3)	0.570 (11)
O1'	0.720 (2)	0.5094 (13)	0.7054 (9)	0.183 (8)	0.430 (11)
O2'	0.8668 (17)	0.6337 (11)	0.6228 (5)	0.107 (5)	0.430 (11)
O3'	1.0098 (17)	0.5589 (15)	0.7614 (8)	0.179 (9)	0.430 (11)
O4'	0.7999 (18)	0.7139 (10)	0.7552 (7)	0.085 (4)	0.430 (11)
C1	1.1968 (7)	0.1905 (5)	1.0250 (3)	0.0783 (13)	
H1	1.2412	0.1350	1.0726	0.094*	
C2	1.2802 (7)	0.2279 (6)	0.9589 (4)	0.0815 (14)	
H2	1.3928	0.2041	0.9510	0.098*	
C3	1.1662 (7)	0.3073 (5)	0.9066 (3)	0.0782 (14)	
H3	1.1897	0.3484	0.8551	0.094*	
C4	0.9047 (6)	0.2323 (4)	1.0594 (3)	0.0628 (11)	
C5	0.8791 (8)	0.1159 (5)	1.0957 (3)	0.0760 (13)	
H5	0.9520	0.0468	1.0885	0.091*	
C6	0.7462 (8)	0.1014 (5)	1.1426 (4)	0.0865 (17)	
H6	0.7288	0.0219	1.1681	0.104*	
C7	0.6402 (8)	0.2000 (6)	1.1527 (3)	0.0802 (15)	
H7	0.5488	0.1893	1.1846	0.096*	
C8	0.6672 (7)	0.3159 (5)	1.1158 (3)	0.0674 (11)	
C9	0.8030 (6)	0.3330 (4)	1.0700 (3)	0.0626 (10)	
H9	0.8240	0.4133	1.0468	0.075*	
C10	0.5544 (8)	0.4191 (6)	1.1244 (3)	0.0762 (14)	

### Atomic displacement parameters (Å^2^)

**Table:**

	*U*^11^	*U*^22^	*U*^33^	*U*^12^	*U*^13^	*U*^23^
Ag1	0.0955 (3)	0.0756 (3)	0.0665 (3)	−0.0075 (2)	0.0251 (2)	0.00463 (17)
N1	0.086 (3)	0.074 (2)	0.0521 (19)	−0.016 (2)	0.0166 (18)	0.0037 (17)
N2	0.083 (3)	0.063 (2)	0.0512 (19)	−0.0112 (19)	0.0176 (18)	−0.0030 (16)
N3	0.103 (4)	0.095 (3)	0.084 (3)	−0.004 (3)	0.039 (3)	0.000 (3)
Cl1	0.0891 (8)	0.0551 (6)	0.0545 (6)	−0.0027 (5)	0.0151 (5)	0.0009 (4)
O1	0.158 (10)	0.110 (9)	0.157 (10)	0.023 (7)	0.079 (8)	0.002 (7)
O2	0.254 (15)	0.164 (11)	0.138 (11)	0.034 (10)	−0.087 (11)	−0.050 (9)
O3	0.217 (12)	0.148 (9)	0.187 (12)	−0.070 (8)	0.100 (10)	0.013 (8)
O4	0.096 (4)	0.076 (4)	0.096 (4)	0.022 (3)	0.042 (3)	0.038 (3)
O1'	0.246 (15)	0.191 (13)	0.128 (11)	−0.158 (12)	0.079 (10)	−0.058 (9)
O2'	0.145 (10)	0.127 (9)	0.063 (6)	0.017 (7)	0.052 (7)	0.033 (6)
O3'	0.164 (13)	0.198 (15)	0.135 (11)	0.075 (11)	−0.069 (10)	−0.072 (10)
O4'	0.125 (9)	0.069 (6)	0.058 (5)	0.016 (6)	0.007 (5)	−0.009 (4)
C1	0.091 (4)	0.077 (3)	0.067 (3)	0.000 (3)	0.016 (3)	0.002 (2)
C2	0.082 (3)	0.092 (4)	0.075 (3)	−0.010 (3)	0.026 (3)	−0.016 (3)
C3	0.094 (4)	0.087 (3)	0.059 (3)	−0.025 (3)	0.028 (3)	−0.008 (2)
C4	0.080 (3)	0.063 (3)	0.045 (2)	−0.016 (2)	0.0128 (19)	−0.0027 (18)
C5	0.101 (4)	0.066 (3)	0.061 (3)	−0.011 (3)	0.016 (3)	0.005 (2)
C6	0.115 (5)	0.075 (4)	0.071 (3)	−0.025 (3)	0.021 (3)	0.019 (3)
C7	0.093 (4)	0.091 (4)	0.058 (3)	−0.024 (3)	0.021 (2)	0.011 (2)
C8	0.078 (3)	0.076 (3)	0.048 (2)	−0.014 (2)	0.014 (2)	0.000 (2)
C9	0.084 (3)	0.058 (2)	0.047 (2)	−0.015 (2)	0.016 (2)	0.0004 (18)
C10	0.087 (3)	0.088 (4)	0.058 (3)	−0.016 (3)	0.025 (2)	0.001 (2)

### Geometric parameters (Å, °)

**Table:**

Ag1—N3^i^	2.154 (6)	Cl1—O3	1.463 (7)
Ag1—N1	2.198 (4)	C1—C2	1.367 (7)
Ag1—O4	2.495 (6)	C1—H1	0.9500
Ag1—O4'^ii^	2.609 (6)	C2—C3	1.370 (8)
N1—C3	1.335 (7)	C2—H2	0.9500
N1—N2	1.362 (5)	C3—H3	0.9500
N2—C1	1.340 (7)	C4—C9	1.362 (7)
N2—C4	1.428 (6)	C4—C5	1.384 (6)
N3—C10	1.137 (7)	C5—C6	1.385 (8)
N3—Ag1^i^	2.154 (6)	C5—H5	0.9500
Cl1—O4	1.385 (5)	C6—C7	1.364 (8)
Cl1—O2	1.390 (6)	C6—H6	0.9500
Cl1—O2'	1.391 (6)	C7—C8	1.387 (7)
Cl1—O4'	1.407 (7)	C7—H7	0.9500
Cl1—O1	1.442 (7)	C8—C9	1.396 (6)
Cl1—O1'	1.453 (7)	C8—C10	1.430 (8)
Cl1—O3'	1.455 (7)	C9—H9	0.9500
			
N3^i^—Ag1—N1	147.16 (17)	O4'—Cl1—O3	86.2 (8)
N3^i^—Ag1—O4	105.89 (19)	O1—Cl1—O3	105.4 (6)
N1—Ag1—O4	89.96 (19)	O1'—Cl1—O3	163.3 (7)
N1—Ag1—O4'^ii^	98.4 (19)	O3'—Cl1—O3	70.7 (9)
N3^i^—Ag1—O4'^ii^	110.8 (19)	Cl1—O4—Ag1	123.1 (4)
C3—N1—N2	104.1 (4)	N2—C1—C2	107.5 (5)
C3—N1—Ag1	128.2 (3)	N2—C1—H1	126.2
N2—N1—Ag1	125.2 (3)	C2—C1—H1	126.2
C1—N2—N1	111.2 (4)	C1—C2—C3	105.1 (5)
C1—N2—C4	128.2 (4)	C1—C2—H2	127.5
N1—N2—C4	120.5 (4)	C3—C2—H2	127.5
C10—N3—Ag1^i^	163.0 (5)	N1—C3—C2	112.1 (5)
O4—Cl1—O2	113.8 (6)	N1—C3—H3	123.9
O4—Cl1—O2'	124.4 (6)	C2—C3—H3	123.9
O2—Cl1—O2'	48.6 (6)	C9—C4—C5	121.2 (4)
O4—Cl1—O4'	118.8 (6)	C9—C4—N2	119.8 (4)
O2—Cl1—O4'	117.0 (8)	C5—C4—N2	119.0 (5)
O2'—Cl1—O4'	114.3 (6)	C4—C5—C6	119.3 (5)
O4—Cl1—O1	110.5 (6)	C4—C5—H5	120.3
O2—Cl1—O1	110.3 (7)	C6—C5—H5	120.3
O2'—Cl1—O1	125.1 (8)	C7—C6—C5	120.7 (5)
O4—Cl1—O1'	69.2 (7)	C7—C6—H6	119.6
O2—Cl1—O1'	60.4 (7)	C5—C6—H6	119.6
O2'—Cl1—O1'	106.9 (6)	C6—C7—C8	119.3 (5)
O4'—Cl1—O1'	110.1 (7)	C6—C7—H7	120.4
O1—Cl1—O1'	90.9 (9)	C8—C7—H7	120.4
O2—Cl1—O3'	134.9 (8)	C7—C8—C9	120.8 (5)
O2'—Cl1—O3'	110.7 (7)	C7—C8—C10	119.7 (5)
O4'—Cl1—O3'	108.1 (6)	C9—C8—C10	119.5 (4)
O1—Cl1—O3'	113.0 (8)	C4—C9—C8	118.7 (4)
O1'—Cl1—O3'	106.5 (7)	C4—C9—H9	120.7
O4—Cl1—O3	107.2 (6)	C8—C9—H9	120.7
O2—Cl1—O3	109.2 (7)	N3—C10—C8	178.7 (6)
O2'—Cl1—O3	60.8 (6)		
			
N3^i^—Ag1—N1—C3	−142.6 (4)	N2—C1—C2—C3	0.2 (6)
O4—Ag1—N1—C3	−22.4 (5)	N2—N1—C3—C2	0.2 (6)
N3^i^—Ag1—N1—N2	58.4 (5)	Ag1—N1—C3—C2	−162.3 (4)
O4—Ag1—N1—N2	178.6 (4)	C1—C2—C3—N1	−0.2 (6)
C3—N1—N2—C1	0.0 (5)	C1—N2—C4—C9	144.8 (5)
Ag1—N1—N2—C1	163.1 (3)	N1—N2—C4—C9	−38.0 (6)
C3—N1—N2—C4	−177.7 (4)	C1—N2—C4—C5	−34.9 (7)
Ag1—N1—N2—C4	−14.5 (5)	N1—N2—C4—C5	142.3 (4)
O2—Cl1—O4—Ag1	−88.2 (10)	C9—C4—C5—C6	0.9 (7)
O2'—Cl1—O4—Ag1	−143.1 (8)	N2—C4—C5—C6	−179.4 (5)
O4'—Cl1—O4—Ag1	55.7 (10)	C4—C5—C6—C7	0.5 (8)
O1—Cl1—O4—Ag1	36.5 (9)	C5—C6—C7—C8	−0.4 (8)
O1'—Cl1—O4—Ag1	−46.4 (7)	C6—C7—C8—C9	−1.1 (8)
O3'—Cl1—O4—Ag1	138.0 (15)	C6—C7—C8—C10	178.7 (5)
O3—Cl1—O4—Ag1	150.9 (7)	C5—C4—C9—C8	−2.3 (7)
N3^i^—Ag1—O4—Cl1	−5.6 (7)	N2—C4—C9—C8	177.9 (4)
N1—Ag1—O4—Cl1	−156.5 (6)	C7—C8—C9—C4	2.4 (7)
N1—N2—C1—C2	−0.2 (6)	C10—C8—C9—C4	−177.4 (4)
C4—N2—C1—C2	177.3 (4)		

Symmetry codes: (i) −*x*+1, −*y*+1, −*z*+2; (ii) −*x*+3/2, *y*−1/2, −*z*+3/2.
